# Countermeasures for Hybrid Threats: The Experience of the European Union and Its Member States

**DOI:** 10.1134/S1019331622100033

**Published:** 2022-09-22

**Authors:** D. Yu. Bazarkina

**Affiliations:** Institute of Europe (IE), Russian Academy of Sciences, Moscow, Russia

**Keywords:** hybrid operations, hybrid campaigns, hybrid threats, European Union, European Commission, European Parliament, European Union External Action Service

## Abstract

The methods and tools used by the European Union to counter hybrid threats are identified: from the fight against terrorism to measures aimed at combating economic competitors and political opponents (mainly, to squeeze Russia and China out of European markets). It is concluded that it is not by chance that neither EU institutions nor the research community have worked out a comprehensive definition of operations to combat hybrid threats. A broad understanding of hybrid threats as practically any (depending on the political situation) actions of the opponent serves to justify the application of any counteraction tool. In the fight against global threats such as terrorism, cybercrime, and the spread of false medical data, the EU takes a systemic approach, which makes it possible to assess the level and degree of the convergence of threats to critical infrastructure and the infosphere, as well as the possibilities of counteraction. At the same time, attempts to use economic, legislative, political, and informational tools to achieve one-sided economic, political, and military advantages do not reduce the degree of tension in the EU’s relations with Russia, China, and some other countries, only increasing the number and strength of hybrid threats. This reduces the EU’s ability to achieve strategic autonomy.

Against the backdrop of growing international tension and accusations against state actors of creating hybrid threats for other countries or integration associations, it is topical to analyze the practice of combating hybrid threats in national and supranational structures. The purpose of this article is to identify the methods and tools through which the practice of countering hybrid threats is being implemented in the EU, both with positive goals (such as combating cybercrime) and as part of the ousting of economic competitors (mainly Russia and China) from European markets and political opponents from the infosphere. Achieving this purpose requires answering the following research questions: (1) How are operations to counter hybrid threats defined? (2) How do the EU and its member states carry out operations to combat hybrid threats relevant to third countries, including Russia (in particular, regarding actions of terrorist organizations, cybercrime, etc.)? (3) How does the EU use the approach to countering hybrid threats to explain the measures taken against economic competitors and political opponents (especially in the context of US−China trade wars and US and EU tensions with Russia)?

## HYBRID THREATS, HYBRID CAMPAIGNS, AND HYBRID THREAT COUNTERMEASURES: DESIGNATION ISSUES

Both in theoretical works and in official EU documents, the concepts of *hybrid operations* and *hybrid campaigns* are very closely related to the concept of *hybrid threats*. The line between them is often vague. Thus, F. Hoffman, whose works underlie the theory of hybrid warfare, in works of different years gives almost identical definitions of hybrid warfare [Hoffman, 2007, p. 29] and threats [Hoffman, 2010, p. 444], highlighting the combination of conventional weapons, irregular tactics, terrorism, and criminal behavior on the battlefield. Most likely, this was one of the causes of a certain confusion about the concepts of war and threat both in subsequent scientific publications and in strategic documents, including in the EU. Nevertheless, while Hoffman focuses primarily on the conduct of war (including by nontraditional means), in the later definitions by EU and NATO experts, the concept of hybrid threats is gradually changing (for example, such threats are interpreted as widespread use of traditional and nontraditional means by the enemy to achieve their goals^1^).

The European External Action Service (EEAS) characterizes *hybrid threats* primarily as actions, listing their options from cyberattacks to disruption of energy supplies.^2^ While the understanding of a threat as, for example, a state capable of developing into a military conflict (such an interpretation is present in the Military Doctrine of the Russian Federation^3^) implies the absence of this conflict and is not always associated with a subjective factor (the enemy) alone, a threat as an action always implies active efforts of the enemy, which in this situation ceases to be a suspected one; consequently, countermeasures will be aimed not so much at resolving the situation as at limiting the capabilities of the other side. A stable definition of operations to counter hybrid threats has not yet been developed either; rather, it is formulated from the contrary (the fight against disinformation, cybercrime, foreign interference, etc.). All this makes it difficult to analyze theoretically the fight against hybrid threats, especially when the combination of traditional and nontraditional actions has become a feature of the very system of international relations [Cusumano and Corbe, 2018, p. 6]. Note, however, that in relation to actors such as criminal and terrorist organizations, the assessment of any action as a threat is quite fair due to their inherently antisocial nature.

The term *hybrid campaign*, which has not yet fully taken shape in the scientific literature, is equally difficult to define. The components of a hybrid campaign are information operations (which justifies the use of counterpropaganda as a response), cyberattacks, espionage, actions of proxy structures^4^ (for example, people or organizations that are conductors of the enemy’s propaganda), and economic and political influence and pressure [Mareš, Holzer, and Šmíd, 2020, pp. 39–41]. Both in theoretical works and in the EU’s approach, certain double standards can be traced: hybrid campaigns, actions, or operations are always those of the enemy, while “countering hybrid threats” is the prerogative of the “goodie” [this is noted by Simons, 2021; Fridman, 2018], identifying as the defensive side.

At the same time, the broad interpretation of hybrid threats adopted in the EU serves as a solid justification for initiatives to develop space infrastructure, health care, food security, etc., as branches of ensuring the security of society. Hence, measures to counter hybrid threats are understood as programs to protect critical infrastructure, health, etc. Operations against hybrid threats in such areas imply, for example, the fight against sabotage in food production or misinformation discrediting scientific approaches in medicine; i.e., they provide an objective benefit. Such operations begin to acquire a negative tenor when they are aimed at squeezing out an economic competitor, limiting the opponent’s freedom of speech in the information space, and expanding military blocs.

## POSITIVE EXPERIENCE OF COUNTERING HYBRID THREATS IN THE EU

The experience of the EU in combating hybrid threats is considered using examples from only a few areas (prevention of terrorism, suppression of propaganda, cybercrime, and disinformation related to the coronavirus infection pandemic). Since mid-2020, these threats have increased around the world, and there are predictions artificial intelligence will increasingly be used for criminal purposes [Caldwell, Andrews, Tanay, and Griffin, 2020]. The pandemic and the associated crisis are being used by extremists and terrorists to spread their ideas. At the EU level, a number of agencies are responsible for countering the above threats, including the EU agencies for cooperation in the field of law enforcement (Europol), criminal justice cooperation (Eurojust), and the EU cybersecurity agency (ENISA).

A systematic approach to countering hybrid threats is being implemented in such key areas as the modernization and harmonization of legislation, the blocking of funding channels for the actors of hybrid threats, and the cooperation of supranational EU structures with national authorities (not only of the member states but also of third countries) within the framework of special operations. Technical security solutions are often provided by their developers, private companies. The unquestionable advantage of the system for combating hybrid threats in the EU is the reliance on expert knowledge, for which cooperation is established with as many specialists as possible, not only from security structures but also from the civil sphere, which makes it possible to assess the consequences of the measures taken for different groups and layers of society.

A useful example of coordinating the operational work of law enforcement officers of the EU, its member states, and third countries is EMPACT (European Multidisciplinary Platform for Combating Crime), which brings together specialists from law enforcement and judicial bodies, EU agencies, customs and tax services, and private companies. Europol reports are used within EMPACT by the Commission and President of the Council of the EU to advise Ministers of Justice and Home Affairs, who take the EU’s priorities in the fight against crime for four years as the basis of national operational action plans. EU and nation-state coordinators organize joint police operations, which usually last several days or weeks.^5^ The EMPACT platform has shown its effectiveness, particularly in the fight against cybercrime, including the detention of twelve suspects in cyberattacks against critical infrastructure on October 26, 2021. The operation was attended by officers from Britain, Germany, the Netherlands, Norway, the United States, Ukraine, France, and Switzerland,^6^ which shows the high possibilities for coordinating such work by EU agencies, including those outside it.

Expert networks remain an important tool in the fight against the described group of hybrid threats, in particular, the Radicalization Awareness Network (RAN), created by the EU Commission in 2011 and uniting 6000 practitioners in the member states, working with groups at risk of terrorist recruitment, as well as with active sympathizers of terrorists and extremists.^7^ Network members share positive experiences, particularly in working with young people or using new ICTs. For example, the “Gaming with the Police” project, launched in the Netherlands in 2020, connects police officers with young people from risk groups in online games;^8^ at present, this practice is used by 21 police teams in the country. The Belgian Federal Police shares its practice of interviewing children returned from conflict zones in the Middle East using a special protocol adapted to work with children.^9^ Steps are being taken to disseminate this experience not only through the RAN channels but also on the UN sites.

The cooperation between law enforcement and digital service providers helps create important practical tools to combat hybrid threats, such as the electronic evidence system. The SIRIUS project has been implemented since 2017 jointly by Europol and Eurojust in cooperation with the European Judiciary Network and offers guidance, training, and tools to access data required in criminal investigations and held by online service providers. These tools are available to law enforcement and judicial authorities through a special closed online platform and mobile application.^10^ Europol and Eurojust view the project as an important step in formalizing cooperation between law enforcement and private companies, although its activity is still largely dependent on the goodwill of the latter. The adoption of the Digital Services Act, designed to strengthen the control of online platforms by EU institutions, will accelerate the introduction of mandatory assistance from digital service providers to law enforcement agencies.

Combating hybrid threats means not only stopping the activities of criminal actors but also conducting information campaigns to prepare society for protection against a growing threat or minimizing information and psychological damage from the actions of intruders. The participants in such campaigns are representatives of supranational EU institutions, national governments, media, business, and civil society institutions. Prevalent is the participation of EU agencies in information operations, among which is the Europol-initiated termination of the activities of 21 websites of groups affiliated with Al-Qaeda and the Islamic State, banned in Russia, in October 2021.^11^ The removal of content by Europol remains the main method of countering terrorist propaganda on the Internet. It receives more attention at the official level than, for example, the dissemination of counternarratives, developed primarily by representatives of civil society, or the redirection of search engine users to sites exposing terrorism (a method used by online platforms). In curbing pandemic-related disinformation, the EU is also relying on cooperation with major online platforms, for which the signing of the Code of Practice on Disinformation has made this work mandatory.^12^ In this area, tools to monitor and rank materials using artificial intelligence, the dissemination of counternarratives through the joint efforts of online platforms, governments, and the media are used.

Of course, the agencies also discuss issues of foreign interference in EU affairs, which are currently deeply politicized. Europol refers to the EBU, repeating that state actors are spreading disinformation in an effort to destabilize governance in the European Union,^13^ but the department’s official website does not mention the specific practice of combating foreign interference. More attention is paid to this area by ENISA, the reports of which provide data on state-sponsored cyberthreat subjects;^14^ however, security practitioners generally avoid overly politicized assessments (including in the publications analyzed above).

## HYPERPOLITICIZATION OF COUNTERING HYBRID THREATS

In the practice of countering foreign interference in EU affairs, excessive politicization is clearly manifested, including in economic issues. The clash of business interests and fierce competition are fundamentally inherent in capitalism. However, political elites are now increasingly involved in economic rivalry in the exchange of accusations of hybrid threats. This ultimately affects the quality of life and information and psychological security of citizens. In the process of squeezing Chinese and Russian companies out of the European market under the slogans of combating hybrid threats, one can trace both the economic interest of local and American suppliers and the political interest of the “Atlantists” as part of the European elite (which reduces not only the quality of EU international cooperation but also the ability of European US allies to strengthen real strategic autonomy [Danilov, 2021, p. 19]). Estonia, Latvia, and Lithuania are considered by the Atlantists as the primary object of Russian hybrid influence, which “is due to the continued energy dependence of these countries on Moscow” [Smirnov, 2020, p. 15].

The restriction of foreign influence is being undertaken in the EU using a scheme reminiscent of the set of measures to combat criminal actors, but with a number of differences. Thus, direct legislative bans on the activities of organizations from an opponent country (denied at the official level) can be replaced by bureaucratic obstacles (many stages of contract consideration, risks of vetoing, etc.) and sanctions pressure (even though it reduces the possibility of making weighted solutions [Biscop, 2021, p. 2] beneficial to both parties). Informational and psychological influence on citizens to discredit the opponent is being exerted, just like in the fight against terrorist propaganda, through all possible channels. Public statements by representatives of the leadership of the EU, its member states, and politicians, broadcasting a message about the actions of the opponent country as a hybrid threat, are accompanied by information campaigns by specialized structures (such as the joint EU−NATO Center of Excellence for Countering Hybrid Threats) or through relevant publications. Third parties (business entities, private think tanks, representatives of the scientific community, etc.) are involved not only in assessing the current situation or practical decisions (for example, removing content distributed by an opponent) but also in public politicized statements. Setting the agenda in the media remains an important information lever.

Indicative in this regard are information campaigns involving government bodies and the media carried out against the Russian and Chinese presence in the European market [Seaman, 2021, p. 7]. According to the Swedish Ministry of Defense, “Disinformation and hybrid activities sponsored by state actors such as China and Russia are part of a new normal.”^15^ The European Values Center, a member of the EU strategic communications group for the east, launched the Kremlin Watch project, where the Russian leadership is accused of trying to put pressure on countries economically dependent on Russian energy to force them to support the Nord Stream 2 project [Svárovský et al., 2019, p. 6].

In the EU countries, government agencies distribute publications containing accusations against China of industrial espionage, and the scientific community is almost directly referred to as its nontraditional subject (for example, in a publication of the Federal Office for the Protection of the Constitution of Germany^16^). Chinese companies are becoming targets for reputation attacks [Pashentsev, 2020, pp. 15–16]. All this, of course, has a negative impact on the reputation of not only business but also Russia and China themselves among Europeans. In 2020, the attitude toward Russia was extremely negative in Sweden, Denmark, and the Netherlands (at least three-quarters of the citizens of these countries).^17^ The growth of anti-Chinese sentiment was manifested in protests against the creation of a large Chinese university in the center of Europe [Shishelina, 2021, p. 28].

An example of a combination of legislative and informational measures is the campaign to oust the Chinese telecommunications company Huawei from the EU countries. At the EU level, the Toolbox^18^ and the Report on risk assessment of 5G networks security^19^ indicate the possible interference of third countries in EU affairs if the 5G supplier has strong ties with the government of the country of origin or if the government can put pressure on the supplier in any form. The latter has been the subject of controversy since practically no company is completely free from the influence of the government of its home country. In the case of China, the reason for noncooperation in the EU countries is the National Intelligence Law, according to which Chinese companies must cooperate with the national intelligence service. A number of EU countries have already banned the use of Chinese 5G equipment; an example is Sweden, where telecom operators must exclude it from their infrastructure until 2025.

In Belgium, a scandal involving Huawei erupted in December 2020. The company sponsored an article by lawyer E. Vermulst criticizing the protectionist law on security measures for the implementation of 5G, which was forcing Chinese manufacturers out of the Belgian market. Later, this article, as well as several others, were circulated on Twitter using 14 fake accounts (according to the New York–based online disinformation investigation agency Graphika, their profile pictures were generated using artificial intelligence^20^). Huawei representatives retweeted messages from fake accounts: according to Graphika, Kevin Liu (Huawei’s head of communications in Western Europe) made 60 such retweets within three weeks, and the official Huawei Europe account, 47. The agency admitted that it was impossible to establish who stood behind the incident. Of course, the mere fact that Huawei managers reposted tweets of fake profiles was a rash move. However, despite the uncertainty of the situation, several Belgian civil servants publicly accused Huawei of attacking the government’s reputation.^21^ Thus, the information was introduced into the already prepared infosphere, where the actions of companies from China are used to discredit the country.

Both the media of the EU countries and its government structures provide a platform for politicians who demand further pressure on Russia and Belarus. Certain double standards in the fight against hybrid threats are evidenced by S. Tikhanovskaya’s speech from the rostrum of the European Parliament; Tikhanovskaya de facto openly called for the undermining of public trust (one of the hybrid actions of which the EBU accuses potential opponents) in the Belarusian president, stating the need for the EU to use a “nontraditional” approach by reaching out to Belarusian civil society on the ground,^22^ in particular, by initiating the nonrecognition of the Belarusian authorities.

On November 28, 2021, during the visit of NATO Secretary General J. Stoltenberg to Latvia and Lithuania, President of the EU Commission U. von der Leyen called for closer cooperation between the EU and NATO in the fight against hybrid attacks.^23^ During this meeting, Director of the Joint EU−NATO Center of Excellence for Countering Hybrid Threats T. Tiilikainen accused Russia, China, and Iran of using nontraditional hybrid methods to compensate for the lack of influence at the international level, and the Belarusian government, of deliberately organizing a migration crisis.^24^ Attempts to connect the latter with the movements of the Russian military against the backdrop of accusations against Russia of plans to invade Ukraine are not new in the context of reports about the instrumentalization of migration on the Russian side (for example, in 2015 a number of such publications appeared in the Finnish media [Alenius, 2021]). There is reason to believe that in the EU, in a situation where “interests in the field of migration policy at different levels of government do not always coincide, and conflicts arise” [Potemkina, 2020, p. 109], blaming, first of all, external actors (Belarus and Russia) is used by the part of the European elite that seeks further confrontation.

## CONCLUSIONS

Both in EU institutions and in the research community, there is no succinct definition of operations to combat hybrid threats. At the same time, the understanding of hybrid threats as practically any (depending on the political situation) actions of the opponent serves as a justification for applying countermeasures to the latter.

In the fight against global threats such as terrorism, cybercrime, and the spread of false medical data, the EU takes a systematic approach, where practice is constantly supported by expert knowledge. This makes it possible not only to assess the level and degree of convergence of threats to critical infrastructure and the infosphere, as well as the ability to counter them, but also to develop tactics to combat them constantly, using the latest technical means and mastering the most relevant platforms (evidence of this is the development of a system of electronic evidence, improving the removal of extremist content, monitoring and suppressing cybercrime, and working with young people in their comfort zones, such as online games, etc.). As such initiatives develop, the fight against hybrid threats could become the basis of EU strategic communication as a synchronization of long-term policy and its communication support for internal and external audiences.

At the same time, in politically sensitive issues, such as the fight against foreign interference, a developed system for combating hybrid threats, combining economic, legislative, and political tools with information campaigns, is used in the framework of trade wars and related informational and psychological confrontation. This does not reduce the degree of tension in the EU’s relations with Russia, China, and some other countries, increasing the number and strength of hybrid threats, which can lead to a military threat and reduce the EU’s ability to achieve strategic autonomy.

## Footnotes

1 NATO Allied Command Transformation, 2010. Bi-SC Input to a New NATO Capstone Concept for the Military Contribution to Countering Hybrid Threats, August 25. https://www.act.nato.int/images/stories/events/2010/20100826_bi-sc_cht.pdf. Cited December 15, 2021.

2 European External Action Service, 2021. A Europe that Protects: Countering Hybrid Threats. https://www.dsn.gob.es/sites/dsn/files/hybrid_threats_en_final.pdf. Cited December 7, 2021.

3 See Military Doctrine of the Russian Federation (approved by the President of the Russian Federation on December 25, 2014, no. Pr-2976).

4 In this term, one can notice the influence of the concept of *proxy wars*—conflicts in which a third party pursues its interests without open participation while supporting one of the warring parties (the latter acts as a conductor (“proxy”)) [see Mumford, 2013, p. 40].

5 Eurojust, 2021. *EMPACT leaflet*. https://www.eurojust.europa.eu/empact-leaflet. Cited December 7, 2021.

6 Europol, 2021. *12 targeted for involvement in ransomware attacks against critical infrastructure*. https://www.europol.europa.eu/newsroom/news/12-targeted-for-involvement-in-ransomware-attacks-against-critical-infrastructure. Cited December 7, 2021.

7 European Commission, 2021. *Radicalisation Awareness Network (RAN)*. https://ec.europa.eu/home-affairs/networks/radicalisation-awareness-network-ran_en. Cited December 7, 2021.

8 European Commission, 2021. *Gaming with the police*. https://ec.europa.eu/home-affairs/networks/radicalisation-awareness-network-ran/collection-inspiring-practices/ran-practices/gaming-police_en. Cited December 7, 2021.

9 European Commission, 2021. *Interviews of returnee children*. https://ec.europa.eu/home-affairs/networks/radicalisation-awareness-network-ran/collection-inspiring-practices/ran-practices/interviews-returnee-children_en. Cited December 7, 2021.

10 Europol, 2021. 3rd Annual SIRIUS EU Digital Evidence Situation Report. European Union Agency for Law Enforcement Cooperation, The Hague, Netherlands, p. 9.

11 Europol, 2021. *Germany, the UK and Europol target violent jihadist websites*. https://www.europol.europa.eu/newsroom/news/germany-uk-and-europol-target-violent-jihadist-websites. Cited December 7, 2021.

12 European Commission, 2021. *Reports on June Actions—Fighting COVID-19 Disinformation Monitoring Programme*. https://digital-strategy.ec.europa.eu/en/library/reports-june-actions-fighting-covid-19-disinformation-monitoring-programme. Cited December 7, 2021.

13 Europol, 2020. *Catching the virus cybercrime, disinformation and the COVID-19 pandemic*. https://www.europol.europa.eu/sites/default/files/documents/catching_the_virus_cybercrime_disinformation_and_the_covid-19_pandemic_0.pdf. Cited December 7, 2021.

14 ENISA, 2021. *ENISA Threat Landscape 2021*, European Union Agency for Cybersecurity (ENISA), Attiki, Greece, pp. 16‒23.

15 Regeringskansliet, 2021. *“France and Sweden to further develop defence cooperation*.*”* https://www.government.se/opinion-pieces/2021/09/france-and-sweden-to-further-develop-defence-cooperation/. Cited December 7, 2021.

16 Spionageabwehr, 2021. Chinas neue Wege der Spionage. *Single Point of Contact—SPOC*, [online] 1, pp. 30−34. https://www.verfassungsschutz.de/SharedDocs/publikationen/DE/2021/spoc-wirtschaft-und-wissenschaft-schuetzen.html. Cited December 7, 2021.

17 Huang C., 2021. *International opinion of Russia and Putin remains negative in 2020*. https://pewrsr.ch/3888Ncl. Cited December 7, 2021.

18 European Commission, 2020. *Cybersecurity of 5G networks— EU Toolbox of risk mitigating measures*. https://digital-strategy.ec.europa.eu/en/library/cybersecurity-5g-networks-eu-toolbox-risk-mitigating-measures. Cited December 7, 2021.

19 European Commission, 2019. *Member States publish a report on EU coordinated risk assessment of 5G networks security*. https://ec.europa.eu/commission/presscorner/detail/en/IP_19_6049. Cited December 7, 2021.

20 Graphika, 2021. *Fake Cluster Boosts Huawei*. Graphika Reports. https://graphika.com/reports/fake-cluster-boosts-huawei/. Cited December 7, 2021.

21 Cimpanu C., 2021. *A network of Twitter bots has attacked the Belgian government’s Huawei 5G ban*. https://www.zdnet.com/article/a-network-of-twitter-bots-has-attacked-the-belgian-governments-huawei-5g-ban/. Cited December 7, 2021.

22 Agence Europe, 24.11.2021 (Brussels, Belgium).

23 Agence Europe, 29.11.2021 (Brussels, Belgium).

24 Hybrid CoE, 2021. *On-going hybrid threats against the EU and NATO*. https://www.hybridcoe.fi/news/on-going-hybrid-threats-against-the-eu-and-nato/. Cited December 7, 2021.
